# Rapid and Efficient Stable Gene Transfer to Mesenchymal Stromal Cells Using a Modified Foamy Virus Vector

**DOI:** 10.1038/mt.2016.91

**Published:** 2016-06-07

**Authors:** Nathan Paul Sweeney, Cathy Regan, Jiahui Liu, Antonio Galleu, Francesco Dazzi, Dirk Lindemann, Charles Anthony Rupar, Myra Olga McClure

**Affiliations:** 1 Jefferiss Research Trust laboratories, Department of Medicine, Imperial College London, London, UK; 2 Department of Pathology and Laboratory Medicine, Western University, Ontario, Canada; 3 Department of Biochemistry and Pediatrics, Western University, Ontario, Canada; 4 Department of Haemato-Oncology, King's College London, London, UK; 5 Institute of Virology, Technische Universität Dresden, Dresden, Germany

## Abstract

Mesenchymal stromal cells (MSCs) hold great promise for regenerative medicine. Stable *ex vivo* gene transfer to MSCs could improve the outcome and scope of MSC therapy, but current vectors require multiple rounds of transduction, involve genotoxic viral promoters and/or the addition of cytotoxic cationic polymers in order to achieve efficient transduction. We describe a self-inactivating foamy virus vector (FVV), incorporating the simian macaque foamy virus envelope and using physiological promoters, which efficiently transduces murine MSCs (mMSCs) in a single-round. High and sustained expression of the transgene, whether GFP or the lysosomal enzyme, arylsulphatase A (ARSA), was achieved. Defining MSC characteristics (surface marker expression and differentiation potential), as well as long-term engraftment and distribution in the murine brain following intracerebroventricular delivery, are unaffected by FVV transduction. Similarly, greater than 95% of human MSCs (hMSCs) were stably transduced using the same vector, facilitating human application. This work describes the best stable gene transfer vector available for mMSCs and hMSCs.

## Introduction

Mesenchymal stromal cells (MSCs) are a heterogeneous population of adult cells of mesodermal origin that can be readily isolated from bone marrow or adipose tissue and efficiently expanded *in vitro* as plastic-adherent cells. They contain a proportion of cells capable of differentiating into osteocytes, adipocytes, and chondrocytes under appropriate conditions.^1^ MSCs have been extensively tested in a number of clinical conditions and proved enormous potential.^2^ *In vivo*, tissue-resident MSCs are thought to be recruited to sites of inflammation where they play a regulatory role in wound-healing, immune modulation, angiogenesis, and tissue homeostasis.^3,4^ Inflammation-directed trafficking may still be retained by exogenously administered MSCs, thus making the case for their use as cancer-targeting cells^5,6^ and site-directed immunosuppressive and pro-repair effector cells,^7^ the latter being extensively exploited for a wide-range of diseases, such as rheumatoid arthritis, type-1 diabetes, and neurological diseases such as amyotrophic lateral sclerosis and stroke.^8,9^

Long-term engraftment of MSCs in the central nervous system (CNS)^10,11^ facilitates their use as cellular vectors for neurodegenerative diseases, including Huntington's disease,^12^ Parkinson's disease,^13^ and lysosomal storage diseases,^14^ which are caused by a lysosomal enzyme deficiency. Lysosomal enzymes are tagged with a mannose-6-phosphate to enable their retrieval from the secretory pathway by its binding to the mannose-6-phosphate receptor.^15^ This retrieval is leaky, resulting in some secretion of lysosomal enzyme.^16^ Secreted enzyme is then retrieved by mannose-6-phosphate receptor at the cell-surface. This secrete-and-recapture system can be exploited in gene and cell therapies since enzyme expression in one cell can correct many others. For example, metachromatic leukodystrophy (MLD), one of the most common lysosomal storage diseases, is caused by a deficiency of arylsulphatase A (ARSA) causing storage of sulphatide in oligodendrocytes, Schwann cells, and neurons leading to progressive demyelination and, in its most common form, death by 5 years of age.^17^ Overexpression of ARSA in hematopoietic stem cells can reduce sulphatide storage caused by ARSA deficiency in the CNS of mice,^18^ despite efficacy being dependent on the recruitment of microglia across the blood-brain-barrier. This demonstrates that relatively few cells expressing ARSA could be sufficient to prevent disease. Since MSCs can be safely delivered to humans, intravenously^19^ and directly to the brain,^20,21,22^ a gene and MSC therapy could complement or exceed a hematopoietic stem cell-based approach.

Any vector for gene transfer to MSCs should persist during cell division to allow *ex vivo* expansion. Vectors based on retroviruses, which integrate the vector DNA into the host genome readily achieve this. However, retroviral gene therapy trials have proven that these vectors carry a significant risk^23,24^ due to genotoxicity that is linked to the vectors' integration sites, the presence of strong viral enhancers and/or transcriptional read-through.^25,26,27,28^ Consequently, modern clinically relevant gene therapy vectors are deleted of promoter/enhancer activity from the viral long terminal repeats (LTRs) (termed self-inactivating (SIN) vectors^29^) and employ nonviral physiological promoters to drive transgene expression.^30^ Nonetheless, the commonly used integrating vectors (γ-retroviral or lentiviral) have so far failed to transduce MSCs efficiently while retaining the aforementioned safety features.^31^ Thus, a SIN vector with a physiological promoter that can efficiently transduce rodent and hMSCs would boost combined gene and MSC therapies.

Foamy viruses are nonhuman, apathogenic viruses that form a distinct subgroup of the *Retroviridae*.^32,33^ Self-inactivating foamy virus vectors (FVV) based on the prototype foamy virus (PFV) are well characterized^34,35,36,37,38^ and effective in large animal models of disease.^39,40^ An historical disadvantage of FVVs is that they induced a marked cytopathic effect (CPE) at a high multiplicity of infection (MOI) *in vitro* due to the fusogenic nature of the PFV envelope (Env).^32^ The simian macaque foamy virus envelope (SFV_mac_ Env) has recently been shown to be less fusogenic than that of PFV,^41^ but has not been described in gene transfer.

This paper describes optimization of FVVs for high transduction efficiency and transgene expression in mMSCs and hMSCs. By employing the SFV_mac_ Env, high transduction efficiencies (>95%) in MSCs are achieved from a single-round of transduction by FVV containing the cellular phosphoglycerate kinase (PGK) promoter. Viral promoters or toxic chemicals are not involved and transgene expression is high and stable for at least 10 passages post-transduction. MSC differentiation potential and surface marker expression is preserved after FVV transduction at high MOI, as is the distribution and long-term engraftment of mMSCs delivered directly to the murine brain. Thus, we describe the best existing vector for stable MSC gene transfer.

## Results

**Figure 1:**
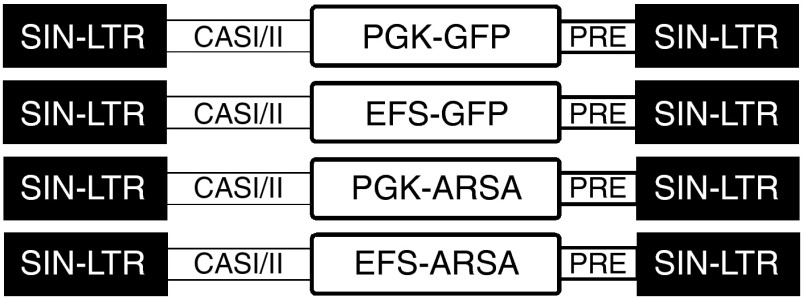
**Schematic of foamy virus vectors (FVVs) used in this study.** All FVVs contain self-inactivating long-terminal repeats (SIN-LTR, black boxes) with the U3 region deleted of promoter and enhancer activity. The cis-acting sequences I and II (CASI/II) are viral sequences necessary for virion assembly. Promoter and transgene of choice are inserted in a multiple cloning site (large white box). A postregulatory element is included in all constructs to improve transgene expression. ARSA, arylsulphatase A (codon optimized for human expression); EFS, elongation factor 1α short (intron-less version); GFP, enhanced green fluorescent protein; PGK, murine phosphoglycerate kinase promoter.

**Figure 2:**
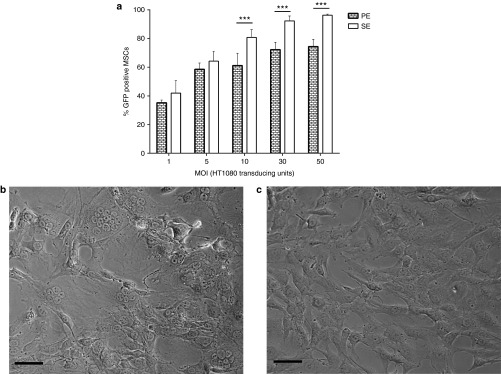
**Effect of foamy virus vector (FVV) envelope on mMSC transduction efficiency and cytopathic effect.** (**a**) The percent of GFP expressing mMSCs after 1 passage post-transduction with PGK-GFP enveloped with PFV Env (brick patterned) or SFV_mac_ Env (white) at different multiplicity of infection (MOIs) was determined by flow cytometry. The mean + SD of biological triplicates is shown. Significant differences between means at each MOI are indicated by asterisks according to the *P* value as determined by two-way analysis of variance with Bonferroni's multiple comparisons test. (**b,c**) Photomicrographs of MSCs 20 hours after vector addition. Scale bar = 50 µm. (**b**) Transduced at MOI 30 using PFV Env; (**c**) Transduced at MOI 30 using SFV_mac_ Env.

The PFV Env induces syncytia formation in target cells at high MOI. Since the less fusogenic SFV_mac_ Env has not yet been tested in gene transfer, we compared the transduction efficiency in mMSCs of FVV with either the PFV or SFV_mac_ Env at different MOIs and assessed the CPE microscopically. Aside from the alternative envelopes, both vectors were identical, each carrying the same PGK-GFP construct (all FVV constructs are shown in **Figure 1**). Both envelopes produced good vector titers, typically ranging between 10^8^ and 10^9^ HT1080 transducing units per ml after 100-fold concentration. Transduction efficiency was determined by flow cytometry as the percent of GFP expressing mMSCs following a single passage post-transduction (**Figure 2a**). At low MOIs of 1 and 5, the transduction efficiency for FVV with either the PFV or SFV_mac_ Env was similar. At higher MOIs of 10 or more, the FVV with SFV_mac_ Env resulted in significantly higher transduction efficiency than with PFV Env. This difference correlated with the MOI at which PFV Env induced extensive CPE, although syncytia formed at all MOIs tested using this envelope. In comparison, no CPE was observed even at the highest MOI of 50 for FVV with SFV_mac_ Env. The highest transduction efficiency by FVV with PFV Env was achieved between MOIs of 30 and 50 with 72 and 74% GFP-expressing cells, respectively, but was accompanied by a marked CPE. At the same MOIs, FVV with SFV_mac_ Env achieved 92 and 95% transduction efficiency, respectively, with no CPE. Representative photomicrographs of mMSCs transduced at MOI 30 with each vector are shown in **Figure 2b**,**c**, demonstrating the widespread syncytia induced only by PFV Env 20 hours after vector addition. All subsequent experiments employed FVV enveloped with SFV_mac_ Env.

**Figure 3:**
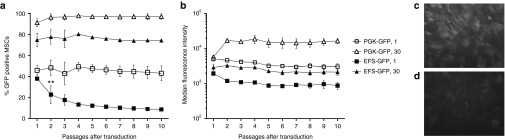
**Comparison of the PGK and EFS promoter for sustained GFP expression in mMSCs.** (**a**) The percentage of mMSCs expressing GFP and (b) their median fluorescence intensity following transduction at multiplicity of infection (MOI) 1 (squares) or MOI 30 (triangles) using FVV:PGK-GFP (white) or FVV:EFS-GFP (black). Flow cytometry analysis was performed when cells reached ~90% confluence at 1 to 10 passages post-transduction. For each passage, 1/10th of the total cells were reseeded. Data points show the mean + SD of data from biological triplicates. Asterisks mark values that are significantly different from the previous passage of the same sample, as determined by two-way analysis of variance with Bonferroni multiple comparisons tests. (**c,d**) Representative photomicrographs of GFP fluorescence in mMSCs transduced at a MOI of 30 with PGK-GFP (**c**) or EFS-GFP (**d**), taken three passages post-transduction. FVV, foamy virus vector.

The PGK and elongation factor 1α short (EFS) promoters are constitutive cellular promoters that, in contrast to viral promoters, have performed well in sensitive genotoxicity assays that measure neighboring gene activation.^30^ The ability of these promoters to achieve high transduction efficiency and maintain expression of GFP in FVV transduced mMSCs through cell expansion, a pre-requisite for cell therapy manufacturing, was compared. Vectors FVV:PGK-GFP and FVV:EFS-GFP (**Figure 1**), were used to transduce mMSCs at MOIs of 1, 30, and 50. Transduced mMSCs were analyzed by flow cytometry to determine the percent of GFP expressing cells (**Figure 3a**) and their median fluorescence intensity (MFI) (**Figure 3b**) after each passage post-transduction up to the 10th passage (cells were passaged once confluence reached over 90% by reseeding one-tenth of the cells). This represents continuous culture over a period of 6 weeks. Representative photomicrographs of GFP fluorescence from MOI 30 transduced mMSCs is shown in **Figure 3c**,**d**. Since a MOI of 50 produced similar results to a MOI of 30 for both constructs at all passages, for clarity only data derived from a MOI of 30 are shown (**Figure 3**).

At a MOI of both 1 and 30, FVV:PGK-GFP transduction results in a higher percentage of mMSCs expressing GFP than FVV:EFS-GFP (**Figure 3a**). Over 96% of mMSCs expressed GFP from two passages post-transduction with FVV:PGK-GFP and was sustained through subsequent passages. Comparatively, transduction with FVV:EFS-GFP at a MOI of 30 (or 50) only resulted in ~75% of mMSCs expressing GFP. At the low MOI of 1, most transduced cells contain a single vector copy (compared to multiple copies at high MOI), allowing for better analysis of expression persistence postexpansion. At this MOI, transduction with FVV:PGK-GFP enabled GFP expression in ~45% of mMSCs, stable over the 10 passages. Conversely, the 40% of mMSCs expressing GFP at 1 passage post-transduction with FVV:EFS-GFP reduced to less than 15% by passage 4 post-transduction. Both EFS and PGK offer stable expression levels in the mMSCs that continue to express GFP, since the MFI does not change after repeated passaging (**Figure 3b**). The PGK promoter drives approximately fivefold higher GFP expression levels than EFS when mMSCs are transduced at a MOI of 30 from the second passage post-transduction, whereas the promoters performed similarly at one passage post-transduction. Together, **Figure 3a**,**b** demonstrates the superiority of PGK as a promoter compared to EFS for FVV-mediated expression of GFP in mMSCs, providing higher expression levels, higher transduction rates, and long-term stability.

**Figure 4:**
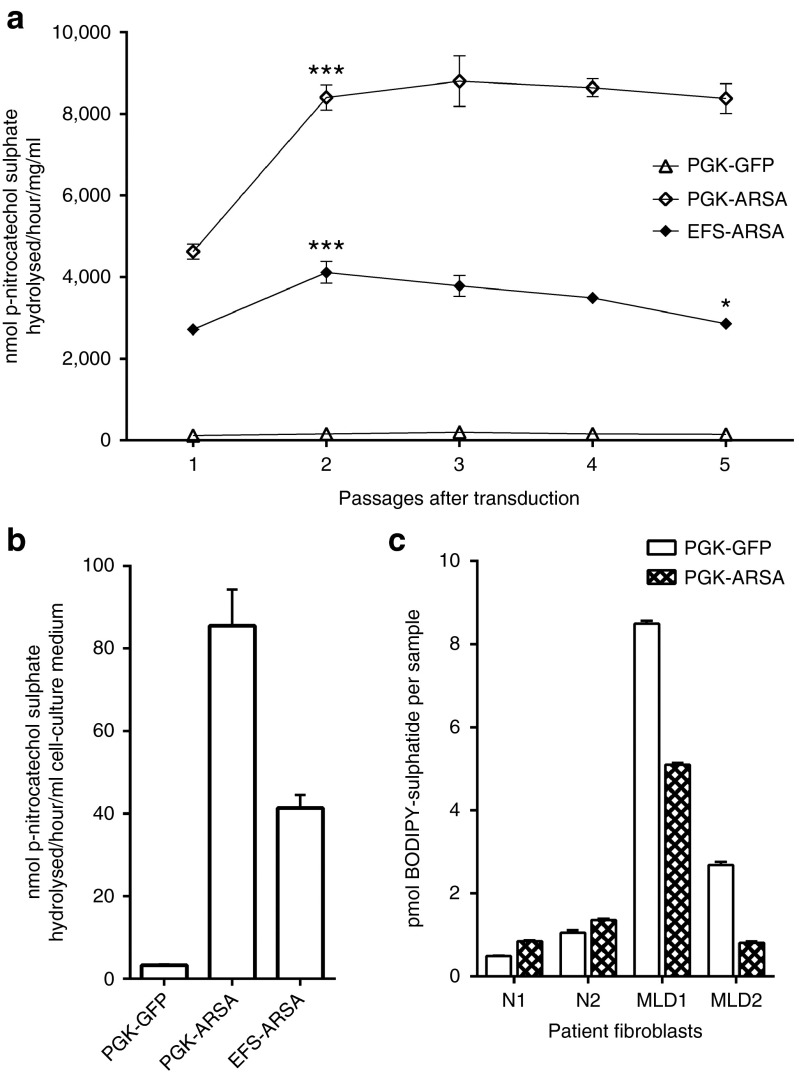
**Foamy virus vector (FVV) mediates high and sustained expression of ARSA in mMSCs.** (a) Lysates of m-MSC transduced cells were taken at each passage from 1 to 5 post-transduction. Lysates were mixed with σ-nitrocatechol sulphate in conditions that specifically enable hydrolysis to be catalyzed by ARSA. The nmol of substrate hydrolyzed per hour and per mg of total protein per ml are given as mean + SD of data from biological triplicates. Two-way analysis of variance with Bonferroni multiple comparisons tests was used to identify significant differences between passages of the same transduced mMSCs. Where a passage was identified to be significantly different from the previous passage of the same population, asterisks are shown to indicate the *P* value. (**b**) The cell-culture medium of mMSCs transduced with the FVV indicated was collected and used in the ARSA assay. The nmol of substrate hydrolyzed per hour per ml of cell-culture medium is given as mean + SD of data from biological triplicates. (**c**) Human fibroblasts from two normal donors (N1 and N2) and two metachromatic leukodystrophy patients' fibroblasts were loaded with BODIPY-sulphatide then cultured in the presence of dilute cell-culture medium from FVV:PGK-GFP (white) or FVV:PGK-ARSA (diamond patterned) transduced mMSCs. After 24 hours, the pmol of BODIPY-sulphatide present in the samples was determined by HPLC analysis. Values (mean + SD of technical triplicates) are normalized to BODIPY-lactosylceramide which was added immediately prior to lipid extraction as a recovery standard.

A codon-optimized arylsulphatase A (ARSA) open reading frame replaced GFP in our FVVs to produce FVV:PGK-ARSA and FVV:EFS-ARSA (**Figure 1**). For high transduction efficiency, mMSCs were transduced at a MOI of 30 with these FVVs and the transduced cells collected after 1 passage. One-tenth of the cells were reseeded until the 5th passage post-transduction. The remaining cells were lysed to determine the intracellular ARSA activity by the ARSA assay^42^ (**Figure 4a**). Low basal activity was detected by mMSCs transduced with FVV:PGK-GFP (control lysate). Both FVV:PGK-ARSA and FVV:EFS-ARSA induced strong ARSA activity, the highest being at passage 2 post-transduction from both vectors. The PGK promoter resulted in a twofold higher enzyme activity at this passage. The ARSA activity remained stable over subsequent passages for FVV:PGK-ARSA-transduced mMSCs, whereas the activity in FVV:EFS-ARSA transduced mMSCs reduced between each passage with a statistically significant reduction between passages 4 and 5 post-transduction. These data are in line with those generated for GFP expression.

Since any MSC-based therapy for a lysosomal storage disease would depend on sufficient enzyme secretion and its correct processing with a mannose-6-phosphate to allow its recapture by endogenous cells, the ARSA activity in mMSC cell-culture medium and its ability to be taken up and used by MLD patients' fibroblasts was determined. The control cell-culture medium from mMSCs transduced with FVV:PGK-GFP had a low ARSA activity (hydrolysing 3.3 nmol of substrate per hour per ml) (**Figure 4b**), whereas FVV:PGK-ARSA-transduced mMSCs hydrolyzed over 80 nmol of substrate per hour per ml, which was twofold higher than FVV:EFS-ARSA transduced mMSCs. Next, the FVV:PGK-ARSA transduced mMSCs cell-culture medium, or that from FVV:PGK-GFP (control medium), was incubated with normal (functional ARSA) or MLD patients' (ARSA deficient) fibroblasts that had been preloaded with fluorescently labelled substrate (BODIPY-sulphatide). Media from both cultures were prediluted to the same extent, such that 0.5 units of ARSA (the amount needed to process 0.5 pmol of substrate per hour in the ARSA assay) was added to fibroblasts in the FVV:PGK-ARSA-transduced mMSC medium. This dilution caused the contribution of endogenous ARSA to be negligible. Fibroblasts from normal donors stored only low amounts of BODIPY-sulphatide in the presence of control or FVV:PGK-ARSA-transduced mMSC medium, as expected (**Figure 4c**). Comparatively, fibroblasts from both MLD patients stored BODIPY-sulphatide in the presence of control medium. Storage was reduced when medium from FVV:PGK-ARSA-transduced mMSCs was added, demonstrating that FVV:PGK-ARSA-encoded ARSA is correctly processed by transduced mMSCs and can correct enzyme-deficient cells.

**Figure 5:**
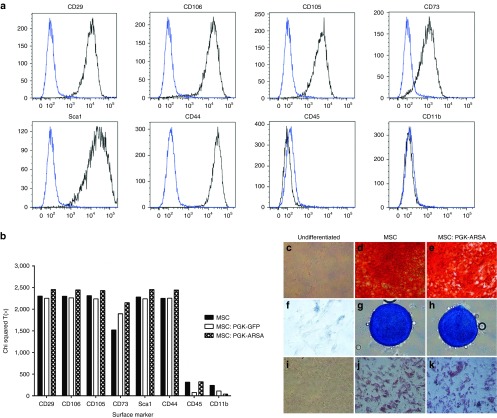
**mMSC characteristics are not perturbed by foamy virus vector (FVV) transduction.** (**a**) A panel of antibodies was used to test surface marker expression on mMSCs transduced or not with FVV. Representative plots (using mMSCs transduced with FVV:PGK-ARSA) are shown comparing isotype control antibody staining (blue) to staining with the antibody indicated above each plot (black). (**b**) The difference between isotype control and surface marker antibody signal intensity was quantified using the FlowJo Chi-squared T(x) comparison and plotted for untransduced MSCs (black), or mMSCs transduced with either FVV:PGK-GFP (white) or FVV:PGK-ARSA (diamond patterned). Higher values of T(x) show greater difference between surface marker antibody and the isotype control. (**c–k**) Representative photomicrographs are shown for mMSCs stained with Alizarin Red (**c–e**), Alcian Blue (**f–h**) or Oil Red O (**i–k**) without inducing differentiation (**c,f,i**) or after culturing in osteogenic (**d,e**), chondrogenic (**g,h**), or adipogenic (**j,k**) differentiation medium. mMSCs were untransduced or transduced with FVV-PGK-ARSA at a MOI of 30 as indicated.

The effects of FVV transduction on mMSC identity and function were examined by comparing untransduced mMSCs and mMSCs transduced at MOI 30 with FVV:PGK-GFP or FVV:PGK-ARSA. A panel of antibodies targeting surface markers known to be expressed or not in mMSCs was employed and staining assessed by flow cytometry. Transduced and untransduced mMSCs stained correctly for all markers with no discernible difference in staining intensity when quantified using the Flowjo Chi squared comparison (**Figure 5a**,**b**). Similarly, transduced mMSCs were able to differentiate into osteocytes, chondrocytes, and adipocytes to a similar extent as untransduced mMSCs when cultured under appropriate conditions (**Figure 5c**–**k**).

**Figure 6:**
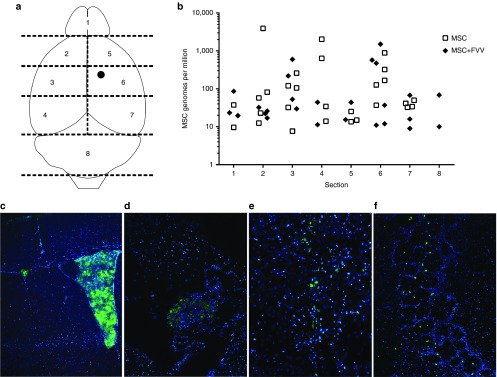
**Foamy virus vector (FVV)-transduced mMSCs maintain their ability to graft in the brains of mice following intracerebroventricular delivery.** (**a**) Sectioning of female mouse brains for qPCR analysis to determine long-term engraftment and distribution of male mMSCs 3 months after the direct delivery of 80,000 mMSCs into the right lateral ventricle (approximate location indicated with solid black circle). The dashed lines indicate the cuts made to produce eight sections (numbered). (**b**) qPCR analysis to quantify mMSCs in the brain sections depicted in a. Each data point represents a section from one mouse. White squares show the engraftment of untransduced mMSCs, whereas black diamonds show the engraftment of FVV transduced MSCs. Data for each group is from six treated mice. Only sections that were above the detection limit are shown. (**c–f**) Photomicrographs showing direct GFP fluorescence in mouse brains following injection of mMSCs transduced by FVV:PGK-GFP. Nuclei, stained with DAPI, are shown in blue. (**c**) Right lateral ventricle immediately postinjection; (**d**) Choroid plexus 45-days postinjection; (**e**) parenchyma showing needle track, 45-days postinjection; (**f**) glomerular layer of olfactory bulb, 45-days postinjection.

Given the prospect of using MSCs for therapy of diseases affecting the CNS, a sensitive quantitative polymerase chain reaction (qPCR) targeting the Y-chromosome to allow detection of male mMSCs delivered directly to the brains of female mice^43^ was established. To determine if FVV transduction affected the long-term engraftment capability of mMSCs, untransduced or FVV:EFS-ARSA-transduced male mMSCs were each injected into the right lateral ventricle of six female mice. Three months postinjection, treated mice were sacrificed and their brains crudely sectioned into eight blocks (**Figure 6a**). Genomic DNA was isolated from each block and the amount of male (mMSC-derived) DNA was determined by qPCR. The number of male genomes present per million total genomes (male and female) is shown for sections 1–8 in **Figure 6b**. Data points are only shown for sections that exceeded the detection limit of approximately 10 male genomes per million. Section 6, which includes the injected ventricle (**Figure 6a**), was the most likely section to contain detectable levels of mMSC DNA with 11 of the 12 injected mice having detectable levels. This section also tended to contain a higher proportion of male DNA than other sections. Section 3, containing the noninjected lateral ventricle, also featured high levels of male DNA in most treated mice. The cerebellum (section 8) was the least likely section to harbor mMSC DNA with only two mice having sufficient numbers for detection, both of which had been injected with FVV transduced mMSCs. Higher numbers of mice had detectable levels of male DNA in all other sections, showing that mMSCs migrate from the injected lateral ventricle throughout the brain. There was no discernible difference in distribution or level of engraftment between transduced and untransduced MSCs.

To test whether FVV-transduced mMSCs could maintain transgene expression *in vivo*, six adult mice were injected in their right lateral ventricle with FVV:PGK-GFP transduced mMSCs. Half the mice were sacrificed immediately postinjection and the other half after 45 days. Coronal cryosections of their brains were examined for direct GFP fluorescence (**Figure 6c**–**f**). Evidence of GFP expression was found for both time-points. The injected lateral ventricle contained many GFP-expressing cells immediately postinjection and was enlarged, while the noninjected ventricle also contained some GFP-expressing cells. After 45-days, GFP-expressing cells were found predominantly, but not exclusively, associated with the choroid plexus; along the needle track route in the parenchyma; and in the glomerular layer of the olfactory bulb. This shows that the PGK promoter remains active in FVV-transduced mMSCs that have grafted long-term in the murine brain.

**Figure 7:**
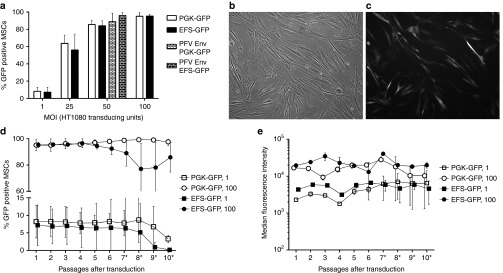
**Human MSCs can be efficiently and stably transduced by foamy virus vector (FVV).** (**a**) hMSCs from three donors were transduced with FVV:PGK-GFP (white) or FVV:EFS-GFP (black) at the multiplicity of infection (MOIs) indicated on the x-axis and the percentage of GFP expressing cells was determined by flow cytometry 1 passage post-transduction. The same vectors, but using the PFV Env, were tested at MOI 50 (brick-patterned bars). (**b,c**) Photomicrographs of hMSCs 3 days post-transduction (no prior passages post-transduction) with FVV:PGK-GFP at a MOI of 100 showing normal cell morphology (**b**) and GFP fluorescence (**c**). (**d,e**) Each time >80% confluence was reached, 1/5th of the hMSCs transduced at MOI 1 (squares) and MOI 100 (circles) were reseeded until 10 passages post-transduction. At each passage, the percentage of GFP expressing cells (**f**) and their median fluorescence intensity (g) was determined by flow cytometry. All panels show the mean + SD of data from biological triplicates. Asterisks in the x-axis labels indicates passages where cultures growth had slowed down, putatively entering senescence (7–10 post-transduction).

To determine whether a FVV-based MSC therapy could be translated from preclinical work in mice to clinical use in humans, the GFP-encoding FVVs were tested on hMSCs obtained from three different donors. At an early passage number (2–4), the hMSCs were transduced with FVV:PGK-GFP or FVV:EFS-GFP at different MOIs. At a MOI of 50, we also tested transduction of these vectors employing the PFV Env. The results (**Figure 7a**) show that, in contrast to results in mMSCs, the PFV Env achieved similar transduction efficiencies to SFV_mac_ Env at high MOI. However, CPE was again induced by PFV Env, although to a lesser extent than in mMSCs at MOI 50 (not shown). At all MOIs and with either envelope, FVV:PGK-GFP and FVV:EFS-GFP perform similarly. For SFV_mac_ Env containing FVVs, under 10% of hMSCs expressed GFP at MOI 1. Each increase in MOI tested resulted in a higher percent of GFP expressing hMSCs. At an MOI of 100, the highest tried, approximately 95% of hMSCs expressed GFP. Normal morphology and no CPE was observed by microscopy at this high MOI (**Figure 7b**,**c**). The average time between passages (one-fifth cells reseeded at each passage) for transduced and untransduced hMSCs was 4 days between each passage until the 7th post-transduction, where the time taken between passages doubled as cells putatively entered senescence. Transduced hMSCs retained normal osteogenic differentiation potential when tested at 6 passages post-transduction (not shown). Therefore, FVV transduction did not affect proliferation or function of hMSCs when assessed by these measures.

Flow cytometry was performed for hMSCs transduced at MOIs of 1 and 100 with both FVV:PGK-GFP and FVV:EFS-GFP following each passage, until the 10th post-transduction, to monitor the percent of GFP expressing cells and their MFI (**Figure 7d**,**e**, respectively). During cell expansion, until growth slowed, neither the percent of hMSCs expressing GFP nor their MFI changed for hMSCs transduced with FVV:PGK-GFP or FVV:EFS-GFP at MOI 1 or 100. Both promoters exerted similar activity. At low MOI, there was variation in transduction efficiency of hMSCs from different donors, but this became unapparent at high MOI. Following the putative entering of senescence, the percent of hMSCs expressing GFP reduced at a MOI of 1 from ~7% presenescence to less than 1% at the 9th passage post-transduction with FVV:EFS-GFP. A reduction was also observed for hMSCs transduced at low MOI with FVV:PGK-GFP. At high MOI, the percent of GFP-expressing cells in FVV:PGK-GFP-transduced hMSCs were unaffected by putative senescence, although the MFI became more variable between hMSCs from different donors. For just one of the three hMSCs transduced with FVV:EFS-GFP at high MOI, the percentage expressing GFP dropped from over 95% presenescence to 50.1% at the 8th passage post-transduction, resulting in the reduction in the average GFP expressing cells transduced with this vector. These data show that, at least presenescence, either of the tested FVVs are highly suited for high and stable transgene expression.

## Discussion

All previous reports on FVVs have used the PFV envelope despite its toxicity to cells at high MOI. The SFV_mac_ Env is less toxic since, in contrast to PFV Env, it only has significant fusion activity at low pH,^41^ thus preventing fusion at the cell membrane. The PFV Env has extremely broad tropism, with only a single zebrafish cell line (Pac2) found to be resistant to PFV infection.^44^ If the SFV_mac_ Env permits equally broad tropism, its lack of toxicity would be advantageous. We have shown that for mMSCs, the SFV_mac_ Env not only allows FVV use at high MOI without inducing syncytia, but unexpectedly enables higher transduction efficiencies to be achieved. Making this simple change may also benefit FVV transduction of other cell types.

We compared the activity of two constitutive cellular promoters, EFS and PGK, in mMSCs and hMSCs over 10 passages. Unexpectedly, the promoter affected the observed transduction efficiency in mMSCs. Since our measure of transduction efficiency depends on detectable GFP expression in each transduced cell, this result is likely caused by the absence of expression in some (FVV:EFS-GFP) transduced cells. This may be due to a combination of the unique integration site selection and/or the inherent heterogeneity within MSC populations. By monitoring expression through cell expansion (**Figure 3**), it becomes apparent that the EFS promoter is subjected to putative silencing, as the percent of GFP-expressing cells decreased during expansion. In contrast, the PGK promoter conferred stable expression which peaked at two passages post-transduction. This late peak may be explained by the FVV's dependence on cell division for integration and transgene expression.^45^ The silencing of EFS may also have played a role in reducing transduction rates using this promoter by counteracting the increase in GFP-expressing cells, since transduction of mMSCs with FVV:EFS-GFP at low MOI did not show a similar peak. Interestingly, neither the expression levels nor the observed transduction rates were influenced in hMSCs by promoter choice (**Figure 7**), indicating different activities of these housekeeping genes between the species of MSCs tested.

Using the lysosomal storage diseases as a proposed target, we showed that ARSA overexpression by FVVs in mMSCs results in strong enzyme activity with a significant amount being secreted. This secreted enzyme was appropriately processed since it could be used by ARSA deficient cells to clear stored substrate. Furthermore, we have shown that mMSC engraftment in the CNS is not affected by FVV transduction and *in vivo* transgene expression is maintained for at least 45 days. Our long-term engraftment levels and distribution were consistent with published results by an independent group using unmodified mMSCs.^43^ Lack of a specific marker to identify MSCs *in vivo* prevents us from determining whether the GFP-expressing (MSC-derived) cells have maintained MSC identity or not. However, for the proposed use of MSCs as cellular vectors, long-term survival and transgene expression is more important than their final identity. Thus, the long-term engraftment and FVV-mediated transgene expression *in vivo* that we demonstrate underpins proposals to use an MSC-based gene therapy approach for the treatment of lysosomal storage diseases affecting the CNS.

Our work has achieved both high and stable transduction efficiency in both mMSCs and hMSCs, allowing the same vector to be employed for preclinical and clinical applications. Importantly, FVV transduction is not enhanced by polybrene,^46^ shown to affect hMSC proliferation capacity.^47^ No additives were used to achieve high transduction efficiency with FVV. Although high transduction efficiency in hMSCs has been reported for lentiviral vectors,^31,48,49,50^ these included viral promoters/enhancers. Even with a viral promoter driving transgene expression, a SIN lentiviral vector required three rounds of transduction to get 92% efficiency in hMSCs.^50^ Given that viral promoters in a retroviral context have been strongly linked to oncogenesis in other cell types,^27,30,51^ these vector designs are unlikely to be approved for clinical use. The vector we describe in this paper does not use viral promoters and, thus, has stronger clinical prospects.

In addition to our vector being devoid of viral promoters, FVVs have innate properties that make them favorable for gene therapy, including a potent transcriptional terminator that prevents transcriptional read-through,^52^ an integration site bias that does not favor active genes or their regulatory regions^53,54^ and being derived from a nonhuman apathogenic virus.^55^ Unfortunately, directly testing our vector's safety in MSCs is challenging. No transformation assays exist for hMSCs which senesce *in vitro* following long-term culture, while mMSCs undergo a pretransformation stage when cultured at ambient oxygen levels *in vitro*, making them unsuitable for such analysis. However, we monitored GFP expression during significant *in vitro* expansion yet saw no indication of clonal dominance, since the MFI and the percentage of GFP-expressing cells were stable in FVV:PGK-GFP-transduced cells (**Figures 3** and **7**). Moreover, hMSCs became senescent at the same passage number in transduced and untransduced cells, indicating that a subpopulation was not transformed. Nevertheless, without sensitive transformation assays available for MSCs, we are unable to draw conclusions on the relative safety of our vector in MSCs. Integration site analysis and testing for oncogenic potential in NOD SCID mice may be appropriate experiments to be carried out prior to clinical translation.

In conclusion, we have developed a potentially safe integrating vector that is highly efficient in both mouse and human MSCs. For both species of MSC, over 95% express transgene stably for at least 10 passages after transduction. All future combined gene and MSC therapies should take advantage of the FVV described in this work.

## Materials and Methods

***Cell isolation and culture.*** All cells were cultured under sterile conditions at 5% CO_2_ and 37 °C in a humidified incubator. The adherent human embryonic kidney and human fibrosarcoma cell lines; HEK-293T^56^ and HT1080; (ref. 57), were cultured in Dulbecco's Modified Eagle's medium containing 10% FBS. mMSCs were isolated from the bone marrow of 4–6-week-old male C57BL/6 mice. Bone marrow was flushed from the femurs and tibias of three mice, pooled and cultured using the Mesencult Mouse Proliferation kit by StemCell Technologies (Cambridge, UK), according to the manufacturer's recommended protocol with the exception that dissociation was performed using TrypLE Express (ThermoFisher Scientific, Hemel Hempstead, Paisley, UK) .

Clinical-grade human bone marrow MSCs were produced in accordance of the Regulation (EC) No 1394/2007 of the European Parliament and of the Council on advanced therapy medicinal products and amending Directive 2001/83/EC and Regulation (EC) No 726/2004. For isolation, 2 ml of bone marrow aspirate was collected from the iliac crests of healthy donors into 100 µl preservative-free heparin. Within 24 hours, cells were seeded at a density of 15–40,000 cells per cm^2^ in alpha minimal essential medium containing antibiotics and 10% FBS. After 3 days, nonadherent cells were discarded and adherent cells cultured until confluence. Cells were maintained using alpha minimal essential medium containing 5% human platelet lysate (Stemulate PL-NH from Cook Regentec, Limerick, Ireland). When cells reached over 90% confluence they were dissociated using TrypLE express and reseeded at a density of 4,000 per cm^2^. All hMSCs were tested for surface marker expression by flow cytometry and satisfied the recommended minimal criteria.^58^ Over 95% were positive for CD105, CD73, and CD90 expression while less than 2% were positive for the negative markers CD45, CD34, CD3, CD14, CD19, and HLA-DR.

***FVV production.*** Generation of transfer vector constructs is described in the **Supplementary Materials and Methods**. FVVs were produced in HEK-293T cells transfected with a 4-plasmid system using PEImax (Park Scientific Ltd., Northampton, UK) at a ratio of 3:1 PEI:DNA. The 4-plasmid system comprised of one of the pDΦ- transfer plasmids, pcoPG4 (encoding PFV Gag), pcoPPwt (encoding PFV pol), and either pcoPE or pcoSE (encoding PFV or SFV_mac_ Env, respectively) in the ratio 52:13:6:4, respectively. Plasmids pcoPG4, pcoPPwt and pcoPE have been described previously.^59^ In a typical transfection, 5 × 10^6^ HEK-293T cells were seeded per 55 cm^2^ round culture dish. The next day, cells were transfected with 15 µg of DNA. Following transfection, published protocols for FVV collection, concentration, and storage^60^ were followed.

***FVV transduction and titration.*** For HT1080 cell transduction, 10^4^ cells were seeded per cm^2^ surface area. After 16–24 hours, FVV was added. After a further 16–24 hours, medium was replaced. For vectors expressing GFP, cells were collected at confluence and the percent of GFP expressing cells (using a vector dilution that gave between 1 and 15% GFP expressing cells) was determined by flow cytometry. The titer was determined by multiplying the proportion of GFP expressing cells by the number of cells at the time of vector addition. For ARSA-encoding vectors, qPCR analysis determined the FVV DNA content 1 passage post-transduction in transduced HT1080 cells relative to that of cells transduced with GFP vector of known titer. For this, the primers 203-F (AGATTGTACGGGAGCTCTTCAC), 203-R (CAGAAAGCATTGCAATCACC) and dual-labeled probe 203-P (FAM-TACTCGCTGCGTCGAGAGTGTACGA-BHQ-1), which target the FVV LTR, were employed. FVV DNA content was normalized to the albumin gene using published primers and probe.^61^

To transduce mMSCs, 2,500 cells were seeded per cm^2^ in the presence of FVV. The cells and vector were centrifuged at 1,200*g* for 90 minutes at 30 °C then cultured normally. After 16–24 hours, medium was replaced. For hMSCs, 4,000 cells were seeded per cm^2^. After 16–24 hours, FVV was added and the cultures centrifuged as for mMSCs. Medium was replaced after 5–8 hours.

***Flow cytometry.*** To assess the percentage of GFP expressing cells and their MFI, at least 10,000 single cells were acquired using a Beckton Dickinson LSRII. Single cells were selected using forward and side scatter parameters. Untransduced cells served as a negative control to set the gates in the 488–530/30 channel to determine the percentage of GFP-expressing cells. For mMSCs, which exhibited strong autofluorescence, the GFP signal in the 488–530/30 channel was plotted against the signal in the 488–610/20 channel. This strategy distinguished between GFP (stronger in 488–530/30 than 488–610/20) and autofluorescence (similar in both channels). Example plots are shown in **Supplementary Figure S1**. The MFI was calculated by subtracting the median signal intensity of the GFP-negative population from that of the GFP-positive population. Surface marker expression analysis for mMSCs was carried out using the Mouse Mesenchymal Stem Cell Marker Antibody Panel (R&D systems, UK) according to the manufacturer's recommended protocol. Data were acquired using a Beckton Dickinson LSRFortessa. Data analysis was performed using FlowJo v10.1 (FlowJo LLC, Ashland, Oregon).

***Functional assays.*** To quantify intracellular ARSA activity, cells were collected 5 days after reaching confluence and lysed on ice in 20 mmol/l Tris-HCl, pH 8.0, 137 mmol/l NaCl, 1% Triton X-100, and 2 mmol/l ethylenediaminetetraacetic acid. Lysates were cleared by centrifugation at 16,000*g* and supernatants collected. Protein concentration was determined using the DC protein assay (Bio-Rad, laboratories Ltd., Hemel Hempstead UK). The ARSA assay was performed as previously described^42^ using between 1 and 2 µg protein per reaction. To determine ARSA activity in cell-culture medium, mMSCs (two passages post-transduction) were grown to confluence and complete medium change was performed. After 5 days, the medium was collected and filtered through a 0.45 µm cellulose acetate syringe filter and stored at −20 °C until use. For each reaction, 40 µl medium was added. All reactions were performed in triplicate or quadruplicate in a 96-well microtiter plate.

BODIPY-sulphatide was produced using Lysosulphatide (Matreya, State College, Pennsylvania), BODIPY FL C16 (Life Technologies, Mississauga, Canada) and dicyclohexylcarbodiimide (Sigma-Aldrich, Ontario, Canada) as previously described.^62^ Patient fibroblasts (MLD or healthy controls) were grown to 75% confluence and BODIPY-sulphatide was added to a final concentration of 6.7 mmol/l. After 24 hours, cells were rinsed twice with PBS and diluted MSC cell-culture supernatant added. Cells were collected for high-performance liquid chromatography analysis after a further 24 hours. BODIPY-lactosylceramide (synthesized as described for BODIPY-sulphatide) was added as a recovery standard and lipids were extracted using chloroform and methanol as described.^63^ Chromatographic separations were performed using a Luna C18 column (Phenomenex; Torrance, California). The mobile phase consisted of solvent A: methanol:water (1:1; v/v) and solvent B: tetrahydrofuran:methanol (4:1; v/v). The flow rate was 1 ml/minute. When eluting the column, the mobile phase was increased from 40% solvent B to 100% solvent B in 20 minutes, held at 100% solvent B for 10 minutes, then decreased back to 40% solvent B and held for 10 minutes. The fluorescence detector was set with an excitation wavelength of 502 nm and emission wavelength of 530 nm.

The differentiation potential of mMSCs into osteocytes, chondrocytes and adipocytes was tested using StemPro Osteogenesis Differentiation Kit, StemPro Chondrogenesis Kit (both ThermoFisher Scientific) or MesenCult Adipogenic Stimulatory Supplements, mouse (Stemcell technologies), respectively. For hMSCs, osteogenic differentiation was tested as for mMSCs. Manufacturers' protocols were followed in all instances. Differentiation was confirmed by staining with Alizarin Red, Alcian Blue or Oil Red O (all from Sigma-Aldrich, Dorset, UK), respectively.

***Stereotaxic injections and tissue processing.*** ARSA^-/-^ mice, which contain a large deletion in the ARSA gene,^64^ had previously been bred onto a C57BL/6 background at Western University Ontario, Canada. Procedures were carried out in compliance with the guidelines set by the Canadian Council for Animal Care. Stereotaxic injections were carried out as previously described.^65^ Following euthanasia, brains were collected and submerged in RNAlater (Qiagen, Ontario, Canada), then frozen at −80 °C. Brains were either sectioned using a cryostat for microscopy or cut into blocks for genomic DNA extraction. Genomic DNA was extracted using the QIAamp DNA mini kit (Qiagen, Manchester, UK). The amount of male DNA present in female brains was determined by qPCR according to published protocols.^43^ The standard curve was generated by adding genomic DNA from mMSCs to genomic DNA extracted from female ARSA^-/-^ mouse brains.

***Microscopy.*** Photomicrographs of tissue sections were acquired using with the Openlab imaging software (Perkin Elmer, Ontario, Canada) connected to an inverted fluorescence Leica DM IRB microscope (Leica Microsystems, Ontario, Canada). Fluorescence and light microscopy of cells *in vitro* was performed using a Nikon Eclipse TE-2000s and images were captured using the Nikon ACT-1 software (Nikon, Kingston upon Thames, UK).

***Statistical analyses.*** Graphing and statistical analyses were carried out using GraphPad Prism version 6.07 (Graphpad software, San Diego, California). Asterisks denote the *P* value from statistical tests (detailed in Figure legends) where *P* < 0.05*, *P* < 0.01**, *P* < 0.001***.

**SUPPLEMENTARY MATERIAL** **Figure S1.** Flow cytometry to determine percent of GFP expressing mMSCs. **Materials and Methods**
