# Supplementary material for: Rapid and Efficient Stable Gene Transfer to Mesenchymal Stromal Cells Using a Modified Foamy Virus Vector
Source: Mol Ther. 2016 Jun 7;24(7):1227–36. doi: 10.1038/mt.2016.91 (PMC4982542; doi:10.1038/mt.2016.91)
Supplement: Supplementary Figure [file mt201691x1.pdf]

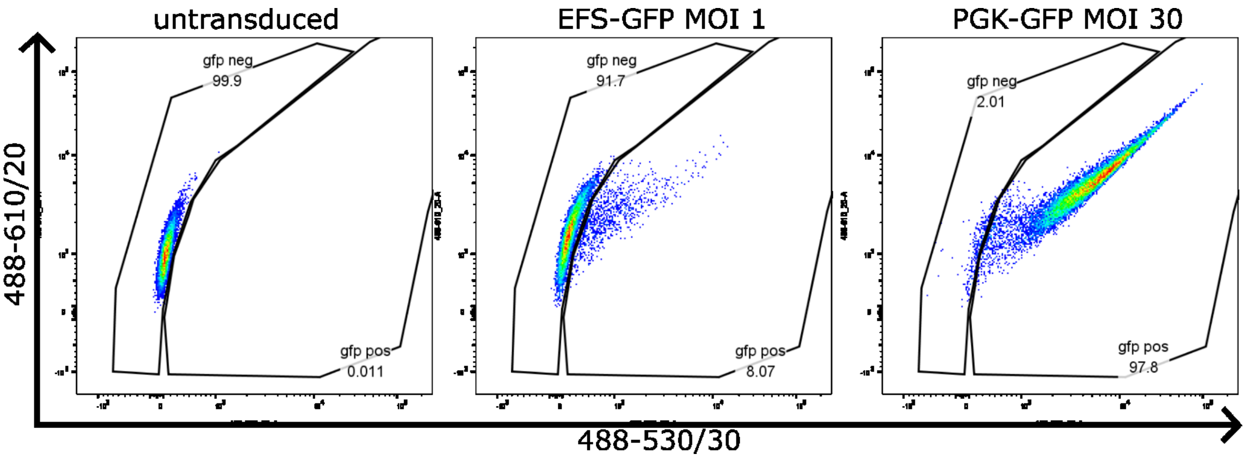

Figure S1 – Flow cytometry to determine percent of GFP expressing mMSCs. FlowJo plots of single cells in the 488-530/30 versus 488-610/20 channels are shown for untransduced mMSCs, FVV:EFS-GFP transduced at MOI 1 (showing the weakest GFP signal), and FVV:PGK-GFP transduced at MOI 30 (showing the strongest GFP signal) as indicated. The GFP negative and GFP positive gates were set around the untransduced mMSC population and applied to transduced samples to determine the percentage of GFP expressing mMSCs, as described in Materials and Methods.
