# Supplementary material for: Rapid and Efficient Stable Gene Transfer to Mesenchymal Stromal Cells Using a Modified Foamy Virus Vector
Source: Mol Ther. 2016 Jun 7;24(7):1227–36. doi: 10.1038/mt.2016.91 (PMC4982542; doi:10.1038/mt.2016.91)
Supplement: Supplementary Materials and Methods [file mt201691x2.doc]

# Supplementary Materials and Methods

**Construction of FVV transfer plasmids**The FVV transfer plasmids were modified from pDΦ^34^, a gift from D. Russell. The murine PGK promoter was PCR amplified from pQ-PGK-FLAG-puro (a gift from G. Maertens) using primers PGK-F (GCATCGATTTCTACCGGGTAGGGGAGGC) and PGK-R (GCGGTACCAGGTCGAAAGGCCCGGAGATG). The EFS promoter was PCR amplified from the EF1α promoter in pWPT-GFP (a gift from J. Luban) using primers EFS-F GCATCGATTGGCTCCGGTGCCCGTCAGT and EFS-R (GCGGTACCCGCGTCACGACACCTGTGTT). Both promoters were inserted between the ClaI-KpnI restriction sites of pDΦ. The enhanced GFP open-reading frame was PCR amplified from pWPT-GFP using primers GFP-F (GCGGTACCATGGTGAGCAAGGGCGAGGA) and GFP-R2 (GCGCGGCCGCAAGCTTCTAGCTACTAGCTAGTCGAG) and cloned into the PCR4-TOPO vector using the Zero Blunt TOPO PCR Cloning Kit for Sequencing (ThermoFisher scientific). The woodchuck hepatitis virus post-transcriptional regulatory element (PRE) was PCR amplified from pLVx-EF1α-IRES-mCherry (Clontech, UK) using primers WPRE-F (GCAAGCTTAATCAACCTCTGGATTACAA) and WPRE-R (GCGCGGCCGCCAGGCGGGGAGGCGGCCCAA) then inserted into PCR4-TOPO containing GFP between HindIII and NotI restriction sites. GFP-PRE was added between KpnI-NotI restriction sites to generate plasmids pDΦ-PGK-GFP-wPRE and pDΦ-EFS-GFP-wPRE. A codon optimised ARSA open-reading frame was amplified from pJO4-ASA (DNA2.0, Menlo Park, CA) using primers ARSA-F (GCATCGATGGTACCATGGGTGCGCCCAGATCGTT) and ARSA-R (GCGGATCCTCACGCATGCGGGTCCGGAC) and inserted between the KpnI and BamHI restriction sites of pDΦ containing EFS or PGK. The optimised PRE^66^ was amplified from pENTR-L5-oPRE-L2 (Addgene plasmid 32414) using primers oPRE-F (GCGGATCCTATACAAAAGTTGTGGAGCA) and oPRE-R (CAGCGGCCGCACGACAACACCACGGAAT) then inserted between BamHI and NotI restriction sites to generate pDΦ-PGK-ARSA-oPRE and pDΦ-EFS-ARSA-oPRE. Sequencing confirmed the correct insert for all plasmids.

66. Schambach, A, Bohne, J, Baum, C, Hermann, FG, Egerer, L, von Laer, D et al. (2006). Woodchuck hepatitis virus post-transcriptional regulatory element deleted from X protein and promoter sequences enhances retroviral vector titer and expression. Gene Ther 13: 641–645.
